# Effect of a Resveratrol/Quercetin Mixture on the Reversion of Hypertension Induced by a Short-Term Exposure to High Sucrose Levels Near Weaning and a Long-Term Exposure That Leads to Metabolic Syndrome in Rats

**DOI:** 10.3390/ijms21062231

**Published:** 2020-03-23

**Authors:** Vicente Castrejón-Téllez, Mariana Villegas-Romero, María Esther Rubio-Ruiz, Israel Pérez-Torres, Elizabeth Carreón-Torres, Eulises Díaz-Díaz, Verónica Guarner-Lans

**Affiliations:** 1 Department of Physiology, Instituto Nacional de Cardiología “Ignacio Chávez”, Juan Badiano 1, Sección XVI, Tlalpan, Mexico City 14080, Mexicoesther.rubio@cardiologia.org.mx (M.E.R.-R.); 2 Department of Cardiovascular Biomedicine, Instituto Nacional de Cardiología “Ignacio Chávez”, Juan Badiano 1, Sección XVI, Tlalpan, Mexico City 14080, Mexico; israel.perez@cardiologia.org.mx; 3 Department of Molecular Biology, Instituto Nacional de Cardiología “Ignacio Chávez”, Juan Badiano 1, Sección XVI, Tlalpan, Mexico City 14080, Mexico; juana.carreon@cardiologia.org.mx; 4 Department of Reproductive Biology, Instituto Nacional de Ciencias Médicas y Nutrición “Salvador Zubirán”, Vasco de Quiroga 15, Sección XVI, Tlalpan, Mexico City 14000, Mexico; eulises.diazd@incmnsz.mx

**Keywords:** hypertension, resveratrol/quercetin, eNOS, oxidative stress, sucrose, critical window near weaning, sirtuins

## Abstract

Hypertension is an important global public health problem. Excess sucrose during a short period near weaning (short sucrose period, SSP; sucrose during rat postnatal days 12 to 28) increases the risk of developing hypertension during adulthood and sucrose ingestion for 6 months after weaning also results in metabolic syndrome (MS) accompanied by hypertension. The aim of this study was to test if the mechanisms that lead to hypertension induced by SSP and MS are similarly modified by a resveratrol/quercetin mixture (RSV/QSC) that targets epigenetic cues. We studied the reversion of hypertension by an RSV/QSC mixture administered for 1 month (from month 6 to month 7 of age) in these two models, since it is effective against some signs of MS. RSV/QSC might determine Sirtuin 1 (SIRT1) and Sirtuin 3 (SIRT3) expression that modulates the expression of endothelial nitric oxide synthase (eNOS), which synthesizes nitric oxide (NO), and of superoxide dismutases (SOD1 and 2), which are antioxidant enzymes that have an impact on the NO levels. Short- (SSP) and long-term (MS) exposure to sucrose induced hypertension and RSV/QSC reversed it. It increased the insulin sensitivity, which may determine the eNOS expression. eNOS expression was decreased in aortas from SSP and MS rats and RSV/QSC only elevated its levels in aortas from MS rats. SIRT1 was also only increased in the MS aortas. Hypertension was accompanied by a decrease in total non-enzymatic antioxidant defenses in SSP and MS aortas, which improved with the RSV/QSC treatment. SOD1 expression was not modified by the sucrose treatments, but SOD2 expression was decreased in SSP and MS aortas. The RSV/QSC treatment increased SOD1 expression in MS aortas. SIRT3 was not modified by the sucrose or RSV/QSC treatments. In conclusion, SSP and MS lead to hypertension, but MS leads to more possible epigenetically- regulated mechanisms related to high blood pressure that could be targeted by the RSV/QSC mixture. Therefore, treatment has better effects on hypertension produced by MS.

## 1. Introduction

Arterial hypertension is considered to be one of the main problems in global public health. It is defined as a systolic pressure above 140 mmHg and a diastolic pressure above 90 mmHg, and it is estimated that 40% of the population aged 25 or over has hypertension. The presence of hypertension increases the risk of developing cardiomyopathies, renal insufficiency, and cerebrovascular accidents [1]. This disease may be related to other pathologies, such as diabetes, metabolic syndrome (MS), and obesity, and several causes predispose the population to develop it, such as the genetic predisposition and unhealthy lifestyles. Sucrose ingestion has been related to the development of hypertension. We have previously reported that sucrose administered during a short period near weaning in rats (postnatal days 12 to 28) (SSP rats) increases the risk of developing hypertension when the organisms reach adulthood [2]. We have also reported that sucrose ingestion for 6 months after weaning in rats results in MS which is accompanied by a high blood pressure [3].

One of the treatments used to improve high blood pressure is caloric restriction and a diet rich in polyphenols, such as resveratrol (RSV) and quercetin (QSC), can diminish blood pressure levels by mimicking the effects of caloric restriction. The phytoalexin RSV (3,4,5-trihydroxystilbene) is found in the skin and seeds of grapes and red wine. RSV protects against diet-induced obesity, insulin resistance, and metabolic diseases [4]. The polyphenolic flavonoid compound QSC (3,5,7,3,4-pentahydroxyflavone) is present in onions, broccoli, tomatoes, apples, and berries, and it has antioxidant, anti-inflammatory, and antiatherogenic properties [5]. RSV and QSC increase the activity of sirtuins (SIRTS), which are a family of histone deacetylases that use oxidized nicotin adenine dinucleiotide (NAD^+^) as a substrate. NAD^+^ is an important energetic sensor in cells [6,7]. The SIRT family is an exciting target for cardiovascular disease management since its members regulate the cardiovascular system in both a direct and indirect manner, by modulating whole body metabolism [8]. Mammals possess seven SIRTs (SIRTs 1–7) that can be found in different subcellular compartments and the level of expression of the different SIRTs varies among tissues. SIRT 1, SIRT 6, and SIRT 7 reside in the nucleus; SIRT 2 is mainly found in the cytosol; and SIRTs 3–5 act in mitochondria [9]. NAD^+^ levels on which SIRTs are dependent are modified by sucrose-rich diets.

We have previously reported a beneficial effect of an RSV/QSC mixture on epigenetic targets, such as SIRTs, in terms of their ability to prevent some of the characteristics of sucrose-induced MS [10]. Regarding the participation of RSV/QSC and SIRTs in the regulation of the development of hypertension, it has been reported that they determine the expression of components related to the nitric oxide (NO) pathway, thus impacting blood pressure. SIRT 1 increases and promotes the activity of coupled endothelial nitric oxide synthase (eNOS) [11,12], and SIRT 3 activates the enzyme superoxide dismutase (SOD), which has an antioxidant capacity. SOD reduces the oxygen reactive species (ROS) that uncouple eNOS and increase NO levels [13]. SIRT3 was found to be decreased at the end of a short period near weaning when rats received sucrose [14].

RSV also increases the GMPc concentration that blocks calcium channels promoting vascular smooth muscle relaxation and promotes an increase in the levels of tetrahydrobiopterine (BH_4_), which is a vasoprotecting coenzyme that elevates the formation of coupled eNOS to produce NO [15].

In this investigation, we studied the reversion, by an RSV/QSC mixture, of hypertension induced by a long-term administration of sucrose that induces metabolic syndrome (MS rats) and by a short period of sucrose (SSP) during a critical window near weaning that also causes hypertension when rats reach adulthood. We evaluated whether the polyphenol mixture might determine SIRT1 and SIRT3 expression modulating eNOS expression and oxidative stress in blood vessels.

## 2. Results

### 2.1. Characteristics of the Experimental Groups

The rats that underwent the long-term sucrose exposure developed MS characterized by increased visceral adipose tissue, insulin, and triglyceride levels, and elevated HOMA-IR. The rats that received sucrose only during the short-term critical window and then received tap water until 7 months of age (SSP) did not exhibit alterations in any of these variables (Table 1).

**Figure 1:**
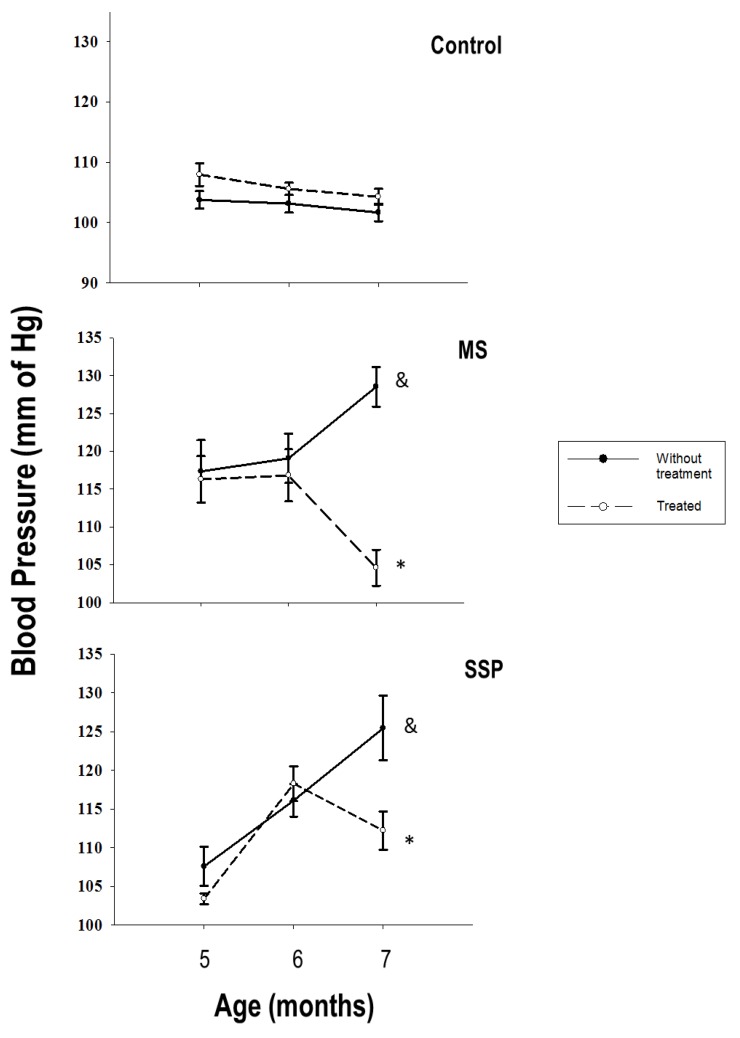
Reversion by treatment for 1 month (from 6 to 7 months of age) with resveratrol (RSV)/quercetin (QSC) of the elevation in blood pressure at 7 months of age caused by exposure to sucrose during the short- (SSP) and long- (MS) term sucrose administration. Data represent the mean ± SEM for six different rats from each group. & *p* < 0.05 against C group without treatment; * *p* < 0.05 against respective group without treatment.

The blood pressure was significantly increased in the two experimental groups when compared to control animals. The RSV/QSC treatment significantly reduced the blood pressure in rats exposed to both the short- and long-term sucrose periods (Figure 1).

**Figure 2:**
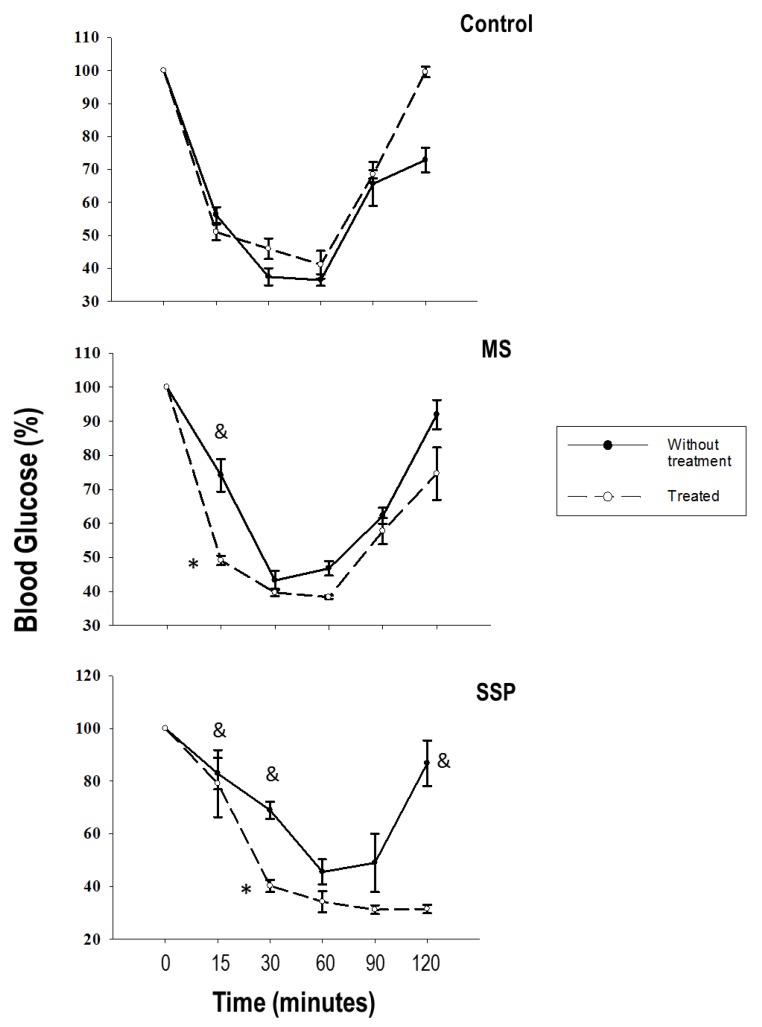
Increased insulin sensitivity caused by treatment for 1 month (from 6 to 7 months of age) with RSV/QSC in rats exposed to short- (SSP) and long- (MS) term sucrose administration. Data represent the mean ± SEM of six different rats from each group. & *p* < 0.05 against C group without treatment; * *p* < 0.05 against respective group without treatment.

We determined the insulin sensitivity and found that the rats that were exposed to sucrose during the short-term glucose exposure (SSP) tended to exhibit more insulin resistance than the rats exposed to long-term sucrose (MS). The RSV/QSC treatment tended to decrease the insulin resistance (Figure 2).

### 2.2. Effect of the RSV/QSC Treatment on eNOS Expression and SIRT1 Expression

**Figure 3:**
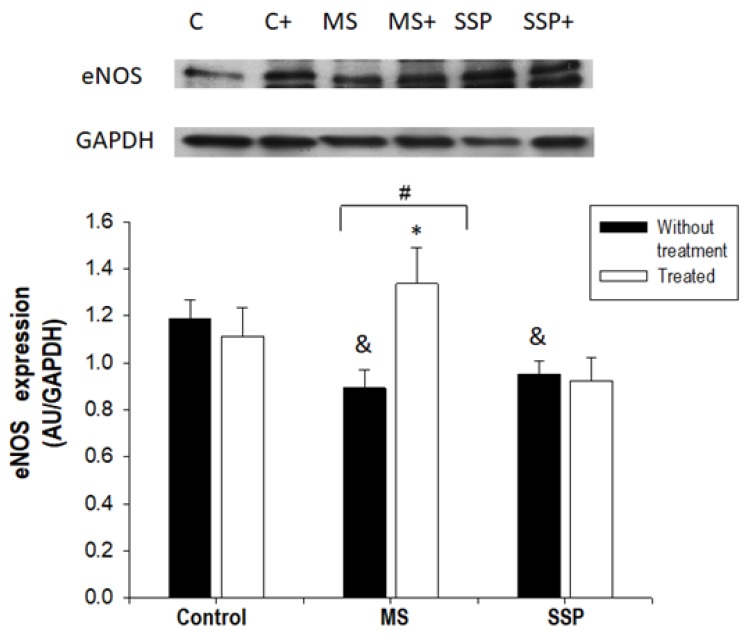
Effect of the treatment for 1 month (from 6 to 7 months of age) with RSV/QSC on the endothelial nitric oxide synthase (eNOS) expression in aorta homogenates after exposure to sucrose for a short- (SSP) and long- (MS) term period. A representative Western blot image is shown. Data represent the mean ± SEM of five different rats from each group. # *p* < 0.01; & *p* < 0.05 against C group without treatment; * *p* < 0.05 against SSP group with same treatment (after one-way ANOVA). Abbreviations: C = control; C+ = control treated with polyphenols; MS = metabolic syndrome; MS+ = metabolic syndrome treated with polyphenols; SSP = short sucrose period; SSP+ = short sucrose period treated with polyphenols.

The expression of eNOS in aortas was significantly decreased by the sucrose treatments. However, the RSV/QSC treatment only significantly increased the eNOS expression in the MS rat aortas (Figure 3).

**Figure 4:**
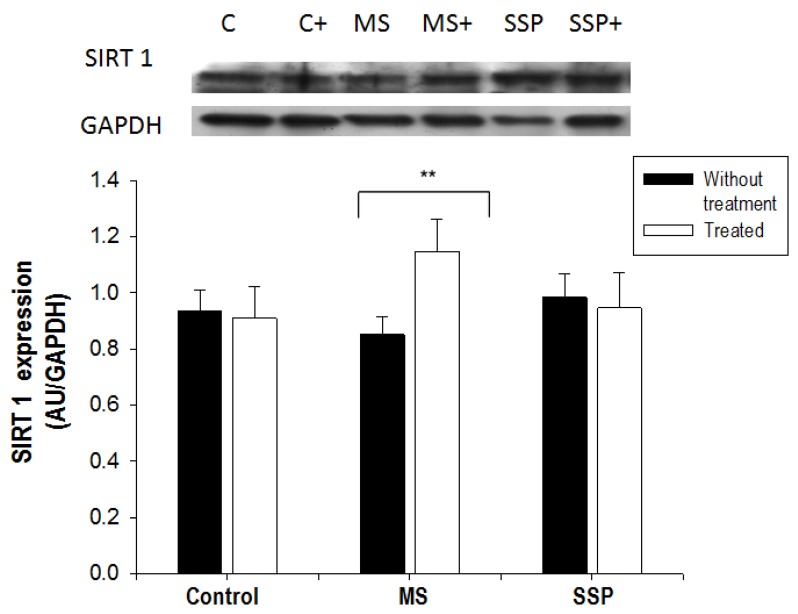
Effect of the treatment for 1 month (from 6 to 7 months of age) with RSV/QSC on the Sirtuin 1 (SIRT 1) expression in rats exposed to sucrose for a short- (SP) and long- (MS) term period. A representative Western blot image is shown. Data represent the mean ± SEM of five different rats from each group ** *p* < 0.05. Abbreviations: C = control; C+ = control treated with polyphenols; MS = metabolic syndrome; MS+ = metabolic syndrome treated with polyphenols; SSP = short sucrose period; SSP+ = short sucrose period treated with polyphenols.

SIRT1 expression, which is an enzyme that regulates eNOS, was not modified by the short- or long-term sucrose treatments. The RSV/QSC treatment did not modify the expression of this sirtuin in the SSP rats, but significantly increased it in the MS rats (Figure 4).

### 2.3. Effect of the RSV/QSC Treatment on Oxidative Stress, SOD1 and SOD2 Expression, and SIRT3 Expression

**Figure 5:**
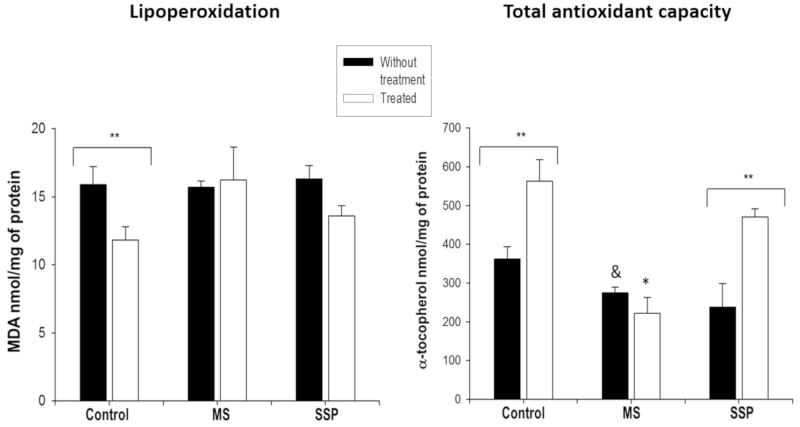
Effects of the short- (SP) and long- (MS) term exposure to sucrose and the treatment with RSV/QSC on lipoperoxidation and the total non-enzymatic antioxidant capacity in rat aortic tissue. Data represent the mean ± SEM of six different rats from each group. ** *p* < 0.05; & *p* < 0.05 against C group without treatment; * *p* < 0.05 against other groups with same treatment (after one-way ANOVA). Abbreviations: C = control; C+ = control treated with polyphenols; MS = metabolic syndrome; MS+ = metabolic syndrome treated with polyphenols; SSP = short sucrose period; SSP+ = short sucrose period treated with polyphenols.

The total non-enzymatic antioxidant capacity was significantly decreased in the aortas of rats exposed to long-term sucrose (MS) and tended to decrease in the aortas from rats exposed to the SSP. The RSV/QSC treatment increased the antioxidant capacity in the control and SSP aortas (Figure 5). Lipoperoxidation was not modified by the sucrose exposures or the RSV/QSC treatment. There was only significant diminution in the control rats, and a tendency of lipoperoxidation to be reduced in the SSP aortas (Figure 5).

**Figure 6:**
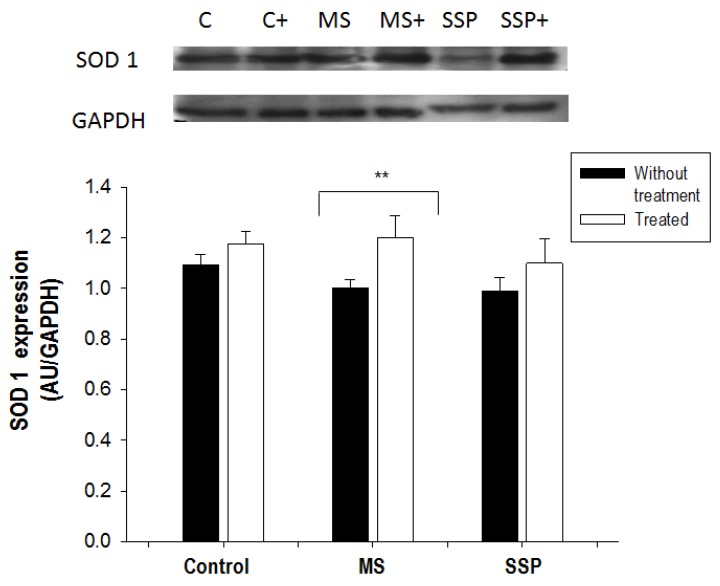
Changes caused by treatment for 1 month (from 6 to 7 months of age) with RSV/QSC of the superoxide dismutase 1 (SOD 1) expression in the aortic tissue caused by short- (SP) and long- (MS) term exposure to sucrose. A representative Western blot image is shown. Data represent the mean ± SEM of six different rats from each group. ** *p* < 0.05. Abbreviations: C = control; C+ = control treated with polyphenols; MS= metabolic syndrome; MS+ = metabolic syndrome treated with polyphenols; SSP = short sucrose period; SSP+ = short sucrose period treated with polyphenols.

There were no significant changes in the expression of SOD 1 with the glucose treatments, but the RSV/QSC treatment significantly increased the expression of this antioxidant enzyme in the MS group (Figure 6).

**Figure 7:**
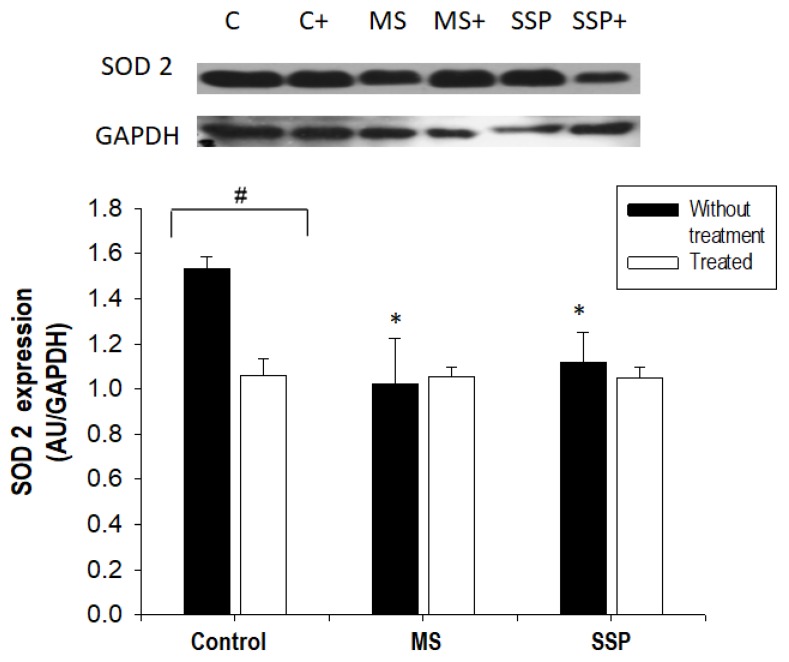
The effects of the administration of RSV plus QSC on SOD 2 expression in the aorta homogenate. A representative Western blot image is shown. Data represent the mean ± SEM of five different rats from each group. # *p* < 0.01; * *p* < 0.05 against C group without treatment (after one-way ANOVA). Abbreviations: C = control; C+ = control treated with polyphenols; MS = metabolic syndrome; MS+ = metabolic syndrome treated with polyphenols; SSP = short sucrose period; SSP+ = short sucrose period treated with polyphenols.

SOD2 was significantly decreased in aortic tissue from the MS and SSP groups (Figure 7). The RSV/QSC treatment only decreased the expression of SOD2 in the control group that did not receive sucrose (Figure 7).

**Figure 8:**
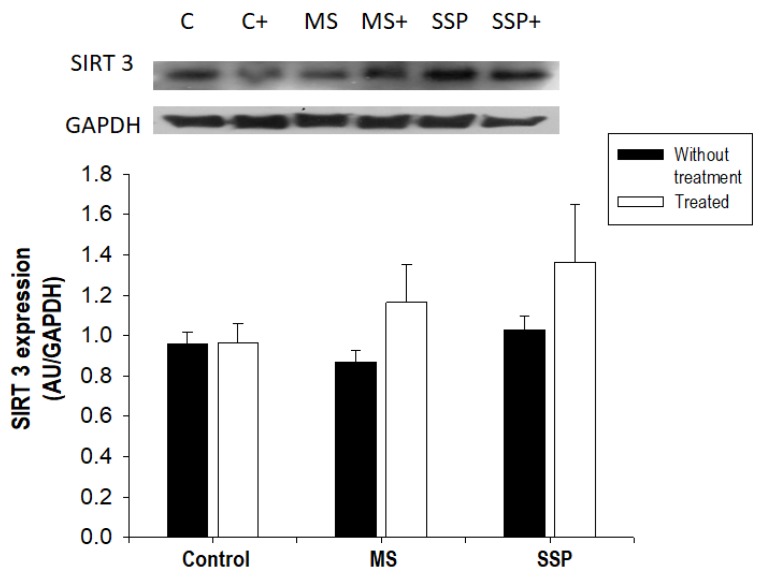
The effects of the long-term (MS) and short-term (SSP) administration of sucrose and polyphenol treatment on the protein expression of SIRT 3. A representative Western blot image is shown. Data represent the mean ± SEM of five different rats from each group. Abbreviations: C = control; C+ = control treated with polyphenols; MS = metabolic syndrome; MS+ = metabolic syndrome treated with polyphenols; SSP = short sucrose period; SSP+ = short sucrose period treated with polyphenols.

The expression of SIRT3, which regulates SOD isoform expression, was not altered by the treatments with sucrose or RSV/QSC. However, the expression of this sirtuin tended to increase in the MS and SSP groups (Figure 8).

## 3. Discussion

In this study, we analyzed the reversion of sucrose-induced hypertension induced by an RSV/QSC mixture in two models that lead to hypertension; long-term exposure that leads to MS [3] and a short sucrose ingestion near weaning that programs the development of hypertension in adults [2]. We studied the mixture’s action on SIRT1 and SIRT3 expression, which modulates eNOS expression and oxidative stress through the regulation of SOD1 and SOD2, respectively. Reversion by this same mixture of polyphenols for signs of MS other than hypertension, such as increased visceral adipose tissue, insulin, triglycerides, and HOMA-IR, which were found in this paper, had been previously reported by our group and other authors [10,16]. Furthermore, perinatal maternal RSV improves the vascular function and prevents the development of hypertension in adult spontaneously hypertensive rat offspring [17].

The blood pressure was increased in both the SSP and MS rats. Hypertension in these groups had already been reported [2,3]. The increase in blood pressure was reversed by the RSV/QSC treatment and the blood pressure decreased in 17% in the SSP group and 11% in the MS group. There are several reports demonstrating that RSV and QSC improve the cardiovascular function in animal models and humans. Chung et al. [18] showed that mice that received a fat- and sucrose-rich diet developed left ventricular hypertrophy, which was reverted by RSV, and that this compound also helped in the post-ischemic functional recovery, reducing the myocardial infarct size. QSC improves the vascular endothelial function. Maccha et al. [19] showed that oral QSC administration (10 mg/kg/day for 4 weeks) to spontaneously hypertensive rats decreased the systolic blood pressure, and the aortic rings from these rats had better endothelial-dependent relaxation induced by acetylcholine and nitroprusiate. QSC metabolites (3-methylquercetin) had endothelium-independent vasodilating effects on porcine mesenteric arteries and the portal and coronary vein. 3-O-β-d-glucoronide QSC also inhibits the growth factor-induced proliferation and migration of vascular smooth muscle cells and decreases angiotensin II-induced hypertrophy [20]. Furthermore, QSC administration (730 mg/day, during 28 days) decreased the blood pressure in hypertensive patients [21].

Investigating the mechanism by which RSV and QSC benefit the function of the cardiovascular system has been the goal of several studies; however, the conclusion that has been reached is that these compounds have pleiotropic effects that are involved in many cellular changes [17]. The prevention of oxidative stress is one of the proposed mechanisms, since RSV and QSC have antioxidant properties [22]. These compounds may also increase the expression of enzymes producing vasorelaxing mediators, such as eNOS, the expression of antioxidant enzymes, and possible epigenetic molecules, including SIRTs.

### 3.1. Effects of the Treatment with RSV/QSC on SIRT 1 and eNOS Expression

In the present study, we found that eNOS expression decreased in the experimental groups that received sucrose for a short- and long-term period. This result is in agreement with findings presented in another paper from our group [2]. Several authors have reported that increases in SIRT1 expression are directly related to the expression and activity of eNOS; however, SIRT1 expression was only increased in the MS aortas in our experiments. Nevertheless, in this study, we did not determine enzyme activities which may lead to the final biological effect of the sucrose or RSV/QSC treatments.

An increase in eNOS after treatment with polyphenols has already been reported [23]. RSV also prevents eNOS uncoupling and enhances eNOS expression [11]. Several in vitro and in vivo studies have proven that RSV induces eNOS expression through the activation of SIRT1, which in turn deacetylates the FOXO transcription factors. Furthermore, SIRT1 deacetylates eNOS in the 496 and 506 lysine residues of the calmodulin binding domain, thus increasing the enzyme’s activity [24,25,26]. SIRT1 also positively regulates the GTPCH 1 enzyme, which mediates a limiting step in the production of the eNOS cofactor, BH_4_ [22,24]. In turn, QSC (5 and 10 µM) in human aortic endothelial cells and C57BL mice aortic rings activates eNOS and mitogen-activated protein kinase (MAPK). It also increases the S-nitrosotioles and nitrites in the culture media. These data suggest that the beneficial effect on endothelial cells might be mediated by MAPK [27].

An increase in eNOS is consistent with the decreased blood pressure found after treatment in MS rats, since an increase in this enzyme’s activity elevates NO production and NO is the main vascular vasorelaxing factor. The increase in SIRT1 found in this study in response to the RSV/QSC treatment is in agreement with the results previously reported by Peredo et al. [10], who observed an elevation in the expression of this protein after 1 month of treatment with RSV/QSC in rat adipose tissue.

RSV and QSC stimulate the expression and activity of SIRTs through the alosteric activation that diminishes the Michaelis constant (Km) for acetylated substrates and NAD^+^ [24,28,29,30]. It has also been found that RSV improves the vascular reactivity and aortic elasticity and diminishes oxidative stress in the vascular wall of elderly and obese mice, and this effect might possibly be due to the direct relation that exists between RSV and the activation of SIRT1 [31,32].

Regarding the insulin sensitivity, we found that there was a tendency of insulin resistance in the SSP group at minutes 15 and 30 after the insulin injection, while in the MS group, this tendency was only observed at minute 15. Insulin could favor hypertension by acting on insulin resistance. This hormone diminishes the activity of the phosphatidyl inositol 3 phosphate (PI3K)/protein kinase B (PKB or AKT)/eNOS pathway. PKB is known to phosphorylate eNOS, thus increasing NO production and vasodilation [33]. The RSV/QSC treatment improved the insulin sensitivity in our SSP and MS group, which is in accordance with the reports from other authors [10,34]. 

### 3.2. Effect of the RSV/QSC Treatment on Oxidative Stress and SIRT3 Expression Which Regulates SOD Expression

Oxidative stress is implicated in the development of cardiovascular diseases, including hypertension [35]. In the present study, the total non-enzymatic antioxidant capacity tended to decrease in the SSP and was significantly diminished in MS aortic tissue. The expression of SOD1 was not modified by the sucrose treatments and SOD2 was decreased in aortic tissue from long- and short-term sucrose-treated rats. From these results, there seems to be an imbalance in ROS production, mainly in the mitochondria (since SOD2 is a mitochondrial enzyme) and their elimination, since the total antioxidant capacity was decreased. This imbalance could induce the uncoupling of eNOS, resulting in an increased production of peroxinitrites, thus diminishing NO synthesis. Therefore, oxidative stress might play an important role in the increased blood pressure in short- and long-term sucrose-induced hypertension. Lipoperoxidation, which is a result of oxidative stress, was not modified by the sucrose treatments when compared to that in the control group without treatment. Nevertheless, the treatment with RSV/QSC tended to reduce the levels of MDA in the SSP group and the treatment significantly increased them in the control group. These results seem to contradict what we had previously reported in other organs in this same model [36]. MDA was elevated in the liver from MS rats and the RSV/QSC treatment decreased it. This discrepancy might indicate that there is a differential response to oxidative stress in each organ, at least in terms of MDA. Therefore, it would be interesting to evaluate other stress markers, such as carbonylation, or the levels of other antioxidants, such as glutathione, in these models with and without treatment with RSV/QSC.

In vitro and in vivo studies have demonstrated that SIRT 3 increases the expression with SOD2 through the activation of FOXO 3 and deacetylation of lysine 68, which increases its activity [37,38]. Although the expression of SIRT3 had been found to be elevated in 28-day-old rats that had received sucrose during the critical window, in our experiments on adult rats (7 months old) that had received sucrose during the critical window, SIRT 3 was not modified by the sucrose treatments, even if SOD2 was decreased. This discrepancy from previous results might be due to random inter-individual variation and might be found if the number of experiments is increased.

After the RSV/QSC treatment, the non-enzymatic total antioxidant capacity was only increased in the control and SSP group. The expression of SOD1 was only increased in the MS. SIRT3 showed a tendency to increase in the MS and SSP groups. These results are in agreement with reports from other groups which indicate that RSV and QSC activate the genes involved in the expression of antioxidant enzymes and the antioxidant capacity [19,22]. RSV regulates the expression of antioxidant enzymes, such as the three SOD isoforms, glutathione peroxidase (GPx), and catalase in cardiovascular tissues through the activation of FOXO 1 and FOXO [26,39]. Previous studies have shown that RSV treatment (50 mg/kg/day) in hepatic mitochondria generates an increase in SIRT3 expression. Other in vitro experiments have shown that low doses of RSV (1–5 µM) directly stimulate the activity of the mitochondrial complex I, elevating the NAD^+^/NADH proportion. The increase in NAD^+^ induces an increase in the tricarboxylic acid cycle and in the oxidation of fatty acids, which are the main mitochondrial substrates [40]. Furthermore, Zhou et al. [41] observed an increase in SIRT3 expression, elevated SOD 2 activity, and a decrease in the expression of acetylated SOD 2 in human umbilical cord cells pre-treated with RSV (10 µM for 2 h). This result can be related to the increase in SOD2 [37].

Some limitations of this study are that we only explored the expression of eNOS and SOD2 and the deacetylases SIRT1 and 3. There may be alterations in the activities of these enzymes that could be participating in the regulation of hypertension independently from the expression.

In summary, short- (postnatal days 12 to 28, SSP) and long- (postnatal day 12 to 7 months, MS) term exposure to sucrose induces hypertension, mediated by a decrease in eNOS expression, in adult rats. The elevated blood pressure in these models is accompanied by an imbalance in oxidative stress caused by a decrease in total non-enzymatic antioxidant defenses and the expression of SOD2. The RSV/QSC treatment reversed the increase in blood pressure and the decrease in eNOS. It also prevented the decrease in the total non-enzymatic antioxidant capacity and SOD2 expression. Although eNOS expression depends on SIRT1, this deacetylase was only modified by the sucrose treatment in the MS group. Even if it has been reported that SOD2 is regulated by SIRT3, we found that it did not continue to be diminished until adulthood. Therefore, SIRT1 and SIRT3 expression could not be the cause of the decreased eNOS and SOD2 expressions. Nevertheless, there could be changes in the activities of these deacetylases, even if their expression did not vary. The RSV/QSC treatment increased SIRT1 and SIRT3 expression independently from their role in the regulation of some the mechanisms that underlie hypertension studied in this paper.

## 4. Materials and Methods

### 4.1. Animals

Experiments were conducted in accordance with the Institutional Ethical Guidelines of the Instituto Nacional de Cardiología “Ignacio Chávez” from Mexico and the protocol was registered at our institution (INCar Protocol number 20-1147, INC/CICUAL/003/20, 23 June 2018). Male newborn rats with a well-known birth date were assigned as a litter of eight pups to each mother on postnatal day 12. For experimental short-term sucrose period rats (SSP), the mother and pups were given 30% sucrose in drinking water (the mother with the eight-pup litter) until day 21, when weaning occurred, and the administration of sucrose was only continued for the pups from day 21 to day 28. The animals were kept under a controlled temperature and 12:12-h light-dark cycle. The rodent commercial food that was provided to the animals contained 23% crude protein, 4.5% crude fat, 8% ashes, and 2.5% added minerals (Lab Diet 5001; Richmond, IN, USA) and was given ad libitum. Control rats received tap water during these days. Tap water was then given to the pups until they reached 7 months of age. At least six rats belonging to three different litters from each group were used. For the long-term sucrose exposure to rats (MS), 30% sucrose in drinking water was given from postnatal day 12 to seven months of age. At six months of age, half of each group of rats (control, MS, or SSP) orally received, in drinking water or sucrose solution, a mixture of RSV and QSC daily for 4 weeks (50 mg/kg/day RSV + 0.95 mg/Kg/day QSC). This dose was chosen after revising the literature to determine the doses that have been proven to have metabolic effects in rats. Furthermore, in some previous studies, we used different doses of this mixture, finding better effects with this dose [10,36]. The mixture of RSV/QSC was provided by ResVitalé and contained 20 mg of QSC per 1050 mg of RSV. The proportions of the compounds provided in commercially available products are chosen according to the proper assessment of absorption/ bioavailability/efficacy and after the study of the distribution between free and bound fractions in blood, which may not necessarily be constant for the different compounds. It has been reported that even if there are relatively small differences in the chemical structures of polyphenols, there are large differences in their binding behaviors towards plasmatic proteins. The hydrophobicity, the presence/absence of some functional groups, steric hindrance, and the spatial arrangement determine the affinity of natural polyphenols towards plasmatic proteins [42]. Furthermore, QSC enhances the bioavailability of resveratrol [43]. This mixture was used instead of the individual compounds since it is sold commercially as a mixture and people are buying and ingesting it, even if the effects are not completely known. Groups without RSV + QSC treatment only received the vehicle in which the natural compounds were dissolved. The mixture of RSV and QSC in ResVitalé capsules was previously dissolved in 1 mL ethanolic solution (20%) [10,36]. The amount of water rats drank was determined daily for 7 days and the dose was calculated according to the volume of water ingested by each animal.

The systolic arterial blood pressure was measured in conscious animals using the tail cuff method, as previously described, when rats reached 5, 6, and 7 months of age [44]. At the end of the experimental period and after overnight fasting (12 h), the animals were killed by decapitation, visceral adipose tissue was obtained and weighed, the thoracic aorta was obtained and cleaned, and blood was collected. Another group of animals was used to determine the insulin sensitivity.

### 4.2. Blood Samples and Biochemical Determinations

Total blood was centrifuged to the separate serum that was then stored at −70 °C until being used for biochemical determinations. An enzymatic SERA-PAKR Plus kit (Bayer Corporation, Sées, France) was used to determine the serum glucose concentration. A commercial radioimmunoassay (RIA) kit specific for rats (Linco Research Inc., Saint Charles, MI, USA) was employed for insulin determination; the sensitivity for the insulin determination of 0.1 ng/mL and the intra- and inter-assay coefficients of variation were 5%, 10%, and 10%, respectively. As a physiological index of insulin resistance, we used the homeostasis model assessment of insulin resistance (HOMA-IR). This index was calculated using the fasting glucose and insulin concentration levels, according to the following formula:

(insulin (µU/mL) × glucose (in mmol/L)/22.5) [45].

Insulin sensitivity was determined by injecting insulin intra-abdominally (1 U insulin/kg diluted in 100 µL saline solution) in fasting rats and then taking blood samples from the tail at 15, 30, 60, 90, and 120 min in conscious animals. Glucose was measured with a glucometer (Abbot, Free Style Optium, UK) using standard reactive glucose strips. 

The total cholesterol (TC) and plasma triglyceride concentrations were measured using commercial enzymatic assays (RANDOX Laboratories Ltd., Crumlin, County Antrim, UK). Plasma was ultracentrifuged at a density of 1.063 g/mL for 2.5 h at 100,000 rpm (Beckman optima TLX) and the bottom fraction was obtained to determine the high-density lipoprotein (HDL) cholesterol content. The difference between the values of TC and HDL-C defines the non-HDL-C in which LDLC, IDL, and VLDL are included. Non-HDL-C is now commonly used as a marker for a blood lipid profile which is associated with an elevated risk of heart disease [2,36].

### 4.3. Thoracic Aorta Homogenization

A sample from the thoracic aorta was taken for homogenization using a lysis buffer (25 mM HEPES, pH = 7.5; 100 mM NaCl, 10% Glycerol, 1% Triton-X100, and 7 mg/mL sodium deoxycholate) supplemented with a mixture of protease inhibitors (1 mM PMSF, 10 µg/mL pepstatin A, 10 µg/mL leupeptin, and 10 µg/mL aprotinin) (Sigma Chemical Co., St. Louis, Missouri, USA). After homogenization of the tissue in liquid nitrogen, it was mixed with lysis buffer at 4 °C. The thoracic aorta homogenate was incubated for 30 min, at 4 °C, in nutator. It was then centrifuged for 10 min at 4 °C at 14,000 rpm. The supernatant was separated and stored at −70 °C until being used. The Bradford method (Protein assay, Bio-Rad laboratories) was used to determine the protein [46]. 

### 4.4. Total Antioxidant Capacity

A volume of 1.5 mL of reaction mixture consisting of the three following solutions, in a relation of 1:10:1 v/v, was used to suspend 100 µL of thoracic aorta homogenate: (a) 20 mM hexahydrate of ferric chloride, (b) 300 mM acetate buffer pH 3.6, and (c) 10 mM of 2,4,6-Tris-2- pyridil-s-triazine dissolved in 40 mM chlorhydric acid. A vortex was used to vigorously shake the mixture for 5 s. Then, the mixture was incubated for 15 min in the dark at 37 ºC. The absorbance was read at 593 nm. Trolox was used to obtain the calibration curve [47].

### 4.5. Immunoblotting of eNOS, SOD1, SOD 2, and SIRT1 and 3

4X loading buffer (20% glycerol, 4% SDS, 0.02% bromophenol blue, 0.2% 2-mercaptoethanol, 125 mM Tris, pH 6.8) was mixed with 50 μg of the aorta homogenate. The mixture was heated for 5 min at 100 °C. SDS-PAGE, bis-acrilamide-laemmli gel was used to separate the proteins using the following concentrations: 8% for eNOS, or 15% for SOD1 and 2. Proteins were then transferred to a 0.22 μm polyvinylidene difluoride (PVDF) membrane. Blots were blocked for 1 h at room temperature using Tris saline buffer plus 0.01% Tween (TBS-T) and 5% non-fat dehydrated milk. Afterwards, membranes were incubated with primary antibodies overnight with a 1:1000 dilution, at 4 °C. The primary antibodies used were rabbit anti-eNOS (sc-376751), mouse anti-SOD1 (sc-271014) and goat anti-SOD2 (sc-18503), rabbit anti-SIRT1 (sc-15404) (all from Santa Cruz Biotechnology, Santa Cruz, CA, USA), and goat anti-SIRT3 (ab118334) (from Abcam, Cambridge, MA, USA). TBS-T buffer was then used to rinse the membranes three times and then they were incubated at room temperature for 3 h with horseradish peroxidase conjugated secondary antibodies at a dilution of 1:10,000 (Santa Cruz Biotechnology, Santa Cruz, CA, USA). All blots were incubated as a control with the GAPDH antibody (sc-365062) (Santa Cruz Biotechnology, Santa Cruz, CA, USA). A chemiluminescence assay (Clarity Western ECL Substrate, Bio-Rad Laboratories, Inc., Hercules, CA, USA) was used for protein detection. X-ray films (AGFA, Ortho CP-GU, Agfa HealthCare NV, Mortsel, Belgium) were used to detect the emitted chemiluminescence. A GS-800 densitometer (including Quantity One software from Bio-Rad Laboratories, Inc.) was employed to acquire the images from each film. We expressed the values of each band density as arbitrary units (AU).

### 4.6. Statistical Analysis

Results were expressed as the mean ± standard error of the mean (SEM) of five different animals per group. For multiple comparisons, we applied one-way analysis of variances (ANOVA) followed by a post hoc Student–Newman–Keuls test, using the program Sigma Plot version 12.3, Jandel Corporation (version 2016, Systat Software Inc., San Jose, CA, USA). For the evaluation of the effects of the RSV/QSC mixture, Student’s t-test was used to determine the differences between the two groups—rats with and without treatment. Differences were considered significant when the *p* value was <0.05.

## 5. Conclusions

Sucrose in both models induced hypertension. The mixture of RSV/QSC reversed hypertension caused by short- and long-term exposure to sucrose by modifying the expression of eNOS, the antioxidant capacity, and the expression of SOD2, and increased the expression of SIRT1 in the aortas of MS rats. In conclusion, the short- and long-term sucrose exposure led to hypertension, but the long-term exposure led to more possible epigenetically- regulated mechanisms related to high blood pressure that could be targeted by the RSV/QSC mixture. Therefore, treatment with this mixture has better effects on hypertension produced by long-term exposure to sucrose, such as that which results in MS.

## Figures and Tables

**Table 1:** Biochemical variables in control, long-term glucose exposure (MS; from postnatal day 12 to 7 months of age), and short-term glucose exposure (SSP; from postnatal day 12 to 28) specimens.

	Control	MS	SSP
Weight (g)	511 ± 4.12	452 ± 6.32	494 ± 5.86
Visceral adipose tissue (g)	4.21 ± 0.61	**9.68** ± 1.23 *	3.98 ± 0.50
Glucose (mg/dl)	115.03 ± 6.85	98.10 ± 6.51	95.4 ± 4.15
Insulin (ng/dl)	6.78 ± 0.61	**14.02** ± 1.07 *	9.39 ± 0.74
HOMA-IR	1.41 ± 0.13	**2.24** ± 0.32 *	1.97 ± 0.21
Triglycerides (mg/dl)	67.16 ± 9.48	**130.05** ± 8.96 *	53.91 ± 5.23
Total cholesterol (mg/dl)	53.07 ± 3.97	51.43 ± 5.03	59.87 ± 4.12
Cholesterol HDL (mg/dl)	27.19 ± 2.03	19.15 ± 1.83	29.98 ± 3.41
Cholesterol non-HDL (mg/dl)	23.31 ± 1.49	31.02 ± 3.89	27.74 ± 2.09

Values represent the mean + SEM of six to eight determinations. Bold and * *p* < 0.05 after a one-way ANOVA test followed by a post hoc Student–Newman–Keuls test.
